# Improvement of Sensitivity and Speed of Virus Sensing Technologies Using nm- and μm-Scale Components

**DOI:** 10.3390/s23156830

**Published:** 2023-07-31

**Authors:** Masato Yasuura, Zheng Lin Tan, Yukichi Horiguchi, Hiroki Ashiba, Takashi Fukuda

**Affiliations:** Sensing System Research Center, National Institute of Advanced Industrial Science and Technology (AIST), Central 5, 1-1-1 Higashi, Tsukuba 305-8565, Ibaraki, Japan; tan.zhenglin@aist.go.jp (Z.L.T.); y-horiguchi@aist.go.jp (Y.H.); h.ashiba@aist.go.jp (H.A.); t-fukuda@aist.go.jp (T.F.)

**Keywords:** virus detection, beads-based assay, digital assay, pore-based assay

## Abstract

Various viral diseases can be widespread and cause severe disruption to global society. Highly sensitive virus detection methods are needed to take effective measures to prevent the spread of viral infection. This required the development of rapid virus detection technology to detect viruses at low concentrations, even in the biological fluid of patients in the early stages of the disease or environmental samples. This review describes an overview of various virus detection technologies and then refers to typical technologies such as beads-based assay, digital assay, and pore-based sensing, which are the three modern approaches to improve the performance of viral sensing in terms of speed and sensitivity.

## 1. Introduction

Globalization has facilitated long-distance travel of people and goods. On the one hand, this promoted progress in technologies and the global economy due to technology transfer; on the other hand, this resulted in the rapid worldwide spreading of viruses. Some of these viruses are pathogenic, and they often cause a global pandemic with serious social disruption. In recent years, we have often heard of the damages due to not only viral infectious diseases that infect humans, such as the recent novel coronavirus infection (COVID-19) [1], severe acute respiratory syndrome (SARS) [2], and Middle East respiratory syndrome (MERS) [3], but also viral infections that infect livestock or agricultural products, such as Avian influenza [4], swine fever [5], and foot-and-mouth disease [6].

Rapid virus detection technology is of great importance in sustaining global economics while preventing the spread of pathological viruses. The detection of viral infection at an early stage helps to contain viruses and limits its spreading, e.g., the early detection of the Ebola virus has limited the spreading of the Ebola virus mainly within West Africa in 2014–2015 [7], while failure to do so is among the reasons which turned both SARS outbreaks into global pandemics. Therefore, the development of highly sensitive rapid virus detection technology is an important proactive measure to construct the safety net to prevent the spread of infection, such as screening and infection risk assessment and management in living spaces, by detecting a very small amount of virus even from asymptomatic people in the early stages of infection and from the environment to cause the contact or airborne transmission [8,9,10].

**Figure 1:**
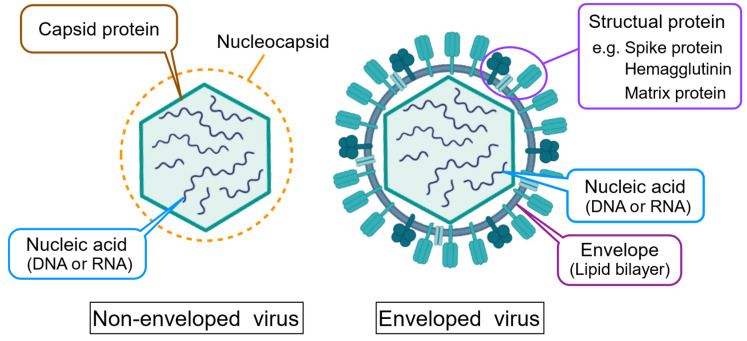
Schematic of viral structures. Whether the virus has an envelope, the virion has specific proteins and nucleic acid sequences.

While virus detection technology is important in the public health and medical sectors, it is a challenging task due to the small size and obligate intracellular functions of viruses [11]. Biological detection methods, such as optical microscopy, are not applicable for virus detection. Conventionally, viruses are detected by a serological approach in which the result of immune response to viral infection, e.g., the production of immunoglobulin, is observed and evaluated. The infectious viral titer assays observe whether the virus can infect cultured cells by conducting either plaque assay [12] or a 50% tissue culture infectious dose (TCID_50_) method [12,13]. The titer assays have the advantage of being able to detect only infectious virions. However, the serological approach for virus detection is cumbersome and low in throughput. Therefore, various virus detection technologies other than the serological approach have been developed to fulfill the need for the rapid and highly sensitive detection of viruses. The principle of these virus detection technologies differs depending on which part of the virus is targeted. Figure 1 shows the schematic structures of viruses. Viruses are classified according to nucleic acid structure (DNA or RNA, single-stranded or double-stranded, linear, circular, segmented, etc.) and morphological properties. As one way of morphological classifying, viruses can be broadly classified into two types: enveloped viruses with an outer membrane (envelope) derived from the lipid bilayer of the host cell, and non-enveloped viruses with exposed capsid proteins [14]. Despite their differences, nucleic acids and proteins are basic components in all viruses, and they are virus-specific, which we can exploit for virus detection. Most rapid and highly sensitive virus detection technologies developed are based on the detection of the molecular fingerprint of viruses.

A representative example of virus detection technology that targets nucleic acids is polymerase chain reaction (PCR), particularly quantitative PCR (qPCR) [15]. This method has become the golden standard for nucleic acid quantitation and has become widely known due to the COVID-19 pandemic. It has been used both as a screening test and a confirmatory test in the diagnosis of viral infection. In addition to qPCR, other methods which allow for nucleic acid amplification at a constant temperature, e.g., loop-mediated isothermal amplification (LAMP) methods [16] and nicking enzyme amplification reaction (NEAR) methods [17], were also used in nucleic acid detection.

On the other hand, another molecular fingerprint of virus, i.e., proteins, were detected and quantified mainly with immunoassays. These assays are also widely known as antigen tests [18,19]. Antibodies that recognize and bind to specific epitopes of viral proteins are used to capture the protein. Following that, another antibody, which was labelled with labelling substances, was bound to either the antigen or capturing the antibody for detection. Immunochromatography [18], for instances, is often used as a rapid test for viral infection based on coloration label. The test usually takes only 10 to 20 min and does not require additional equipment, making it a suitable component for point of care testing. For highly sensitive applications, highly sensitive antigen tests (antigen quantitative tests) [19] are used. This method relies on chemiluminescent label for detection, and the measurement time is 20 to 30 min, while the detection limit is 2 to 3 orders of concentration lower than the rapid test. As this method requires specific equipment for detection, it is generally used in a more professional setting, e.g., in the airport for border control.

**Figure 2:**
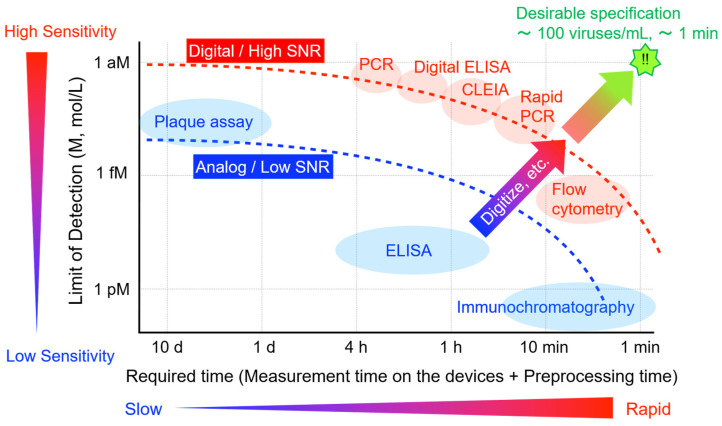
Trade-off relation of the LODs and required times of viral detection methods. For screening and risk management of infections, a breakthrough is desired to overcome the current trade-off relation. The desirable specification for the rapid virus detection is indicated as “!!” in the figure.

In the development of virus detection technologies, progress has aimed mainly at two directions: the reduction of limit of detection (LOD) and the improvement of speed of detection. However, these two directions are in a trade-off relationship, as shown in Figure 2. For example, while PCR has the highest level of sensitivity for virus detection, it is time-consuming (1 to 4 h), and is subject to contamination. On the other hand, the immunochromatographic method can be tested in a short time 10 to 20 min, but the LOD is three to four orders higher than that of PCR. Considering the cases of false negatives, it cannot be used for a definitive negative diagnosis [20]. Recently, nucleic acid-targeted rapid test equipment that can complete the test from sample collection to completion in 10 to 20 min has appeared, but LOD is two to three orders of concentration higher than normal PCR [21]. For the efficient screening of asymptomatic patients, a high-performance method in which the LOD is equivalent to or better than PCR and speed is equivalent to or better than immunochromatography is desirable. A breakthrough is required to overcome the current trade-off relationship between LOD and speed of detection.

This review describes an overview of efforts to improve the LOD and speed of detection of virus detection technologies, with particular attention to three approaches: bioassays using micro- or nanoparticles (bead-based assays), digital bioassays, and pore-based sensing. These approaches have a common characteristic: the use of nm-size or μm-size components, such as magnetic nanoparticles, μm-scale well-arrays, and nm-scale pores.

## 2. Bead-Based Assays for Virus Detection

**Figure 3:**
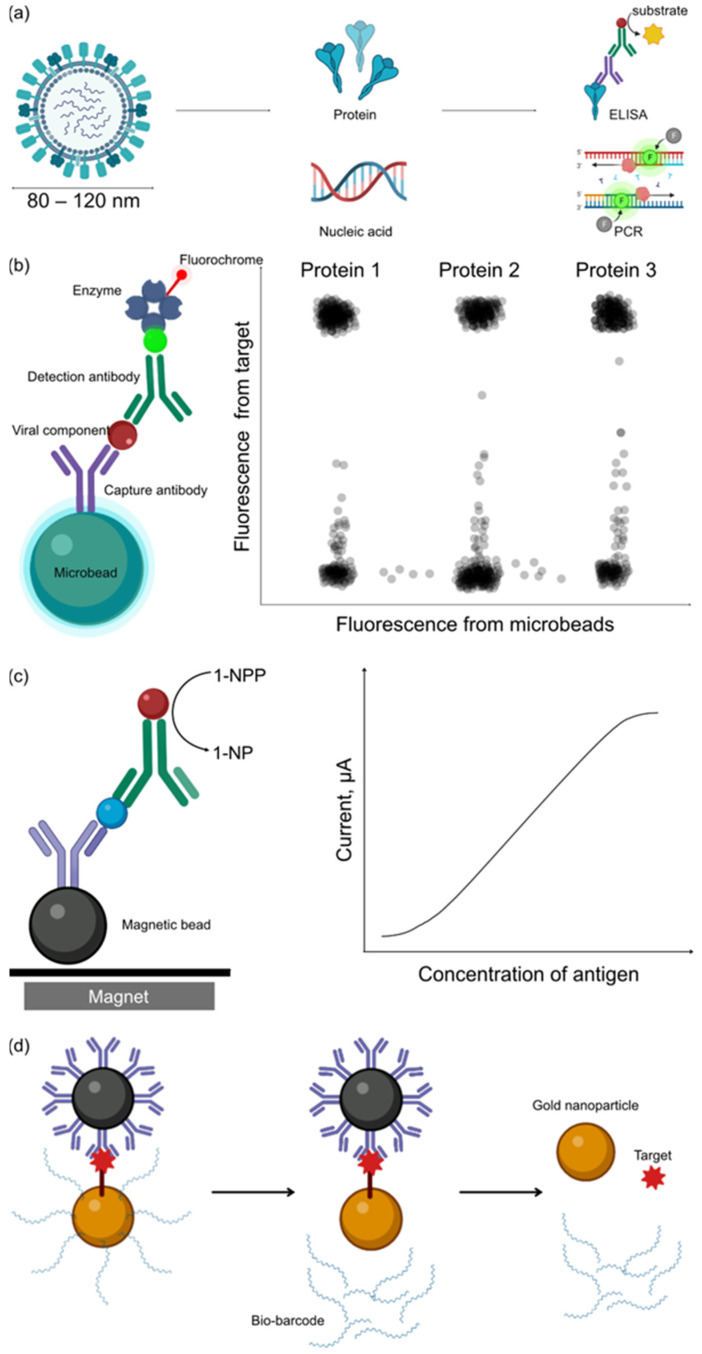
Bead-based assay for virus detection. (**a**) Conventional methods for virus detection. Viruses were usually too small to be detected by direct observation of virus particles. They are generally detected with their proteins via ELISA or nucleic acid via quantitative PCR. (**b**) Virus detection through bead-based ELISA. Multiplex analysis of viruses allows the detection of multiple proteins on viruses or multiple viruses in a single assay. (**c**) ELIME assay. 1-NPP is oxidized into 1-NP, followed by the release of electrons into the reaction buffer. The current produced by the electron is related to the concentration of the antigen. (**d**) Bio-barcode assay. The target is sandwiched between captured microbeads and barcode beads. Next, the microbeads were trapped with a magnet, and barcode DNA was dehybridized. The barcode DNA and nanoparticles will be quantified to investigate the number of targets in each assay.

The characteristics of viruses, as described in the previous section, is a factor which has limited the sensitivity and speed of detection. The small size of virus particles has limited the use of conventional biological detection methods, e.g., microscopic examination to detect virus particles in biological sample; its property with obligate intracellular functions has increased the difficulty for in vitro detection of viruses (Figure 3a). To address these problems, bead-based assays have been proposed for virus enrichment and detection.

Bead-based assays are indirect virus detection methods in which virus particles in biological samples were captured and enriched with microbeads (0.5–500 μm). Following that, the signals generated from these microbeads were detected.

While the concept to apply particles in the biological study was introduced by Wagner et al. in 1964 [22], it was not until after 2004 when Langer and Tirrell proposed methods for surface modification of materials for medicine [23] that bead-based assays were optimized. Today, both magnetic and non-magnetic beads are used for virus detection, with the optical detection of non-magnetic beads the most popular method used, as the signal can be evaluated with equipment available in most biochemical laboratories.

### 2.1. Bead-Based ELISA

For the optical detection of viruses captured by microbeads, captured antibodies will be coated on fluorescence microbeads, e.g., Luminex microbeads. The coating can be achieved either by passive adsorption of antibody on the surface of microbeads, binding through biotin-avidin binding, or by the covalent binding through amine groups. Virus particles are captured by the capture antibody on the surface of microbeads and detected by detection antibody and fluorochrome. The beads–virus complex will be analyzed with flow cytometer by detecting the fluorescence signal emitted from the fluorochrome on detection antibody (Figure 3b). This method can be considered as a form of enzyme-linked immunosorbent assay (ELISA), and each bead represents an independent assay. However, bead-based assay is more efficient and cost-saving than conventional ELISA, as the surface area to volume ratio of sphere is larger than the flat surface used in conventional ELISA, and the free moving microbeads could increase the probability of collision with target molecule. Furthermore, by using two fluorescent dyes in beads and precisely controlling the ratio of dyes, it is possible to conduct multiplex analysis with the bead-based method, which could greatly reduce the sample volume required for analysis, e.g., Khan et al. have demonstrated successful multiplex detection of six target analytes with <1 μL serum [24].

### 2.2. Bead-Based Electrochemical Assay

In addition to optical detection, bead-based detection based on the electrochemical properties of materials was also exploited and used in virus detection. Some enzymes catalyze the oxidation of substrate, in which electrons will be released into its environment after enzymatic reaction. The flow of electrons into a reaction buffer can be measured as a current. By correlating the magnitude of current with the concentration of antigen, we could thereby quantify the concentration of viruses with the magnitude of current. However, the measurement of current in nA–μA magnitude is difficult with distance between analyte and electrode. Although it is possible to adsorb antibodies on electrodes by coating a layer of polymer on an electrode to facilitate the passive adsorption of proteins, the polymer might interfere with the detection of weak currents generated by enzymes when the concentration of the target virus is low. To address this problem, Gehring et al. proposed capturing the target analyte with antibody-coated magnetic nanoparticles and localizing these magnetic nanoparticles on electrodes with a magnet to increase the sensitivity of current detection (Figure 3c). This method is known as enzyme-linked immunomagnetic electrochemistry (ELIME) [25]. Generally, alkaline phosphatase, which could oxidize 1-naphthyl phosphate to 1-naphthol, was used in ELIME for its high stability. As color development or the use of tertiary antibody is not required for ELIME, it could reduce the time of ELISA from 8 h to 2 h. As an example, ELIME assay has been employed in the direct quantification of the hepatitis A virus. The low titer and the undefined structure of proteins from hepatitis A virus [26] render it difficult to be quantified by ELISA. Currently, hepatitis A virus is detected by the presence of hepatitis A virus antibodies, which has prevented the early detection of hepatitis A virus in patients. ELIME assay provided a solution for the direct quantitation of hepatitis A virus between 10^−10^–5 × 10^−7^ IU/mL range with a detection limit of 10^−11^ IU/mL [27]. The results obtained from hepatitis A virus ELIME correlated to results obtained from RT-qPCR, with high coefficient of correlation, suggesting that the quantitation is feasible.

In addition to ELIME, label-free electrochemical bead-based assay, e.g., immunomagnetic impedance metric sensor, was also developed. An antigen-antibody reaction will result in changes in electrochemical impedance, which allows for label-free detection of the target. In this method, first, streptavidin-coated magnetic microbeads are immobilized on the surface of gold electrode with a magnetic bar to ease the process of regeneration of sensing surface when necessary. Next, the biotinylated antibody was coated on magnetic microbeads followed by introduction of the sample. The concentration of target was correlated to changes in electron transfer resistance differences ∆R_m, and ∆R_m is defined as

∆R_m=R_(et(Ab))−R_(et(Ab−Ag))

where R_(et(Ab)) is electron transfer resistance after antibody immobilization and R_(et(Ab−Ag)) is electron transfer resistance after antigen binding to antibody [28].

Other than proteins, nucleic acid can also be captured and analyzed with bead-based assay. Either peptide nucleic acid or a fragment of complementary DNA was immobilized on the surface of magnetic beads to trap target DNA from samples. Magnetic beads were used instead of direct immobilization of complementary DNA on probes or electrodes, as it is difficult for long fragments of DNA to hybridize directly short fragment of nucleic acid attached on the surface of electrodes. After incubation, magnetic beads were collected with magnets, and the non-complementary DNA was removed by washing. Then, intercalators were introduced, the beads were collected after reaction, and the electrochemical signal from the intercalator was measured. This method is known as genomagnetic electrochemical bioassay [29,30]. The limit of detection of genomagnetic electrochemical bioassay depends on the properties of the intercalator. For example, when Meldola’s blue was used as an intercalator, the system had a detection limit of 2 pmol/L, a dynamic range of 2–20 pmol/L, and a hybridization time of 20 min [31].

### 2.3. Bio-Barcode Assay

To further reduce the limit of detection of bead-based method to amol/L order, the bio-barcode method has been proposed [32,33]. The bio-barcode generally consists of two components: (1) magnetic beads bearing probes (either monoclonal antibody or DNA) to capture target; (2) gold nanoparticles bearing detection probes (DNA or polyclonal antibody) and a custom-designed oligonucleotide which will be used to identify the gold nanoparticle. This custom oligonucleotide is also known as bio-barcode.

In bio-barcode assay, first, the target will be captured with magnetic beads. Then, nanoparticles with detection probes and barcode are incubated with magnetic beads–target complexes. Magnetic beads–target–nanoparticles complexes are isolated with magnetic field, and the barcode will be dehybridized from nanoparticles. Either barcode DNA or nanoparticles will be detected (Figure 3d). The amount is correlated to the concentration of the target. Therefore, it can be used to quantify the concentration of target. The limit of detection of bio-barcode assay depends on the choice of components, reagents, and the species of viruses. For example, the limit of detection for hepatitis B virus was 1 pmol/L [34], 100 fg/L for bluetongue virus [35], and 100 pg/L for human immunodeficiency type 1 capsid antigen [36]. Nevertheless, evaluating the limit of detection in ideal setting suggested that the sensitivity of bio-barcode system is almost similar to PCR method, which is approximately 500 zmol/L, or approximately 10 copies in a 30 μL sample [37].

### 2.4. External Force-Assisted Near-Field Illumination Biosensor

In addition to the detection of targets captured by particles, motion-based sensing has also attracted considerable attention due to its potential in real-time detection and high spatial resolution [38,39]. However, it was difficult to control and analyze the motions of many particles in high-resolution in the bulk solution, which resulted in lower sensitivity of these systems than the PCR assay, i.e., 10^3^ virus particles/mL. To address this issue, our group has an External Force-Assisted Near-field Illumination (EFA-NI) biosensor [40,41] that uses magnetic force as the bead driving force, which helps to reduce false positivity by simultaneous detection of optical signals from markers and particle movements.

In and near the evanescent field from near-field illumination, the EFA-NI biosensor uses an external force (magnetic force) to move the target which has bound to antibody-modified magnetic particles. This movement of particles is used to distinguish signal from noise. While various studies have demonstrated the use of magnetic particles for target manipulation, EFA-NI is the first to combine the movement of magnetic particles and optical signal detection to reduce false positivity. Our approach has enhanced the contrast between signal and noise by detecting the “moving optical signal”, thus increasing the signal-to-noise ratio [40,41]. Since the entire space that the near-field illumination reaches becomes the detection area, it is possible to use the concentration effect of the surface. Simultaneously, because of the movement, it is also possible to avoid the influence of noise due to non-specific adsorption on the surface of detection [40]. The EFA-NI biosensor does not require a washing process, and even samples containing many contaminants can be measured simply by mixing the sample with a detection solution (including antibody-modified magnetic particles and optical markers) and injecting it into the device [40,41].

**Figure 4:**
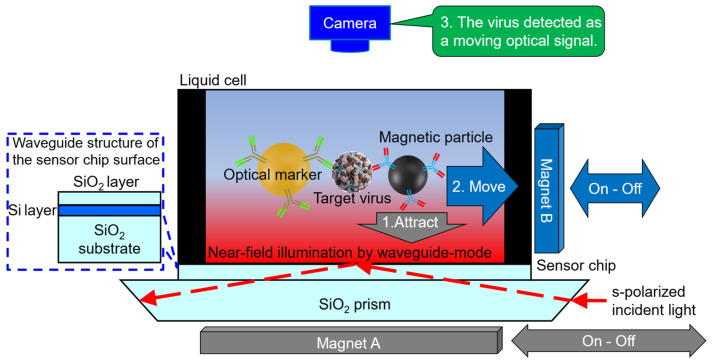
Schematic diagram of the EFA-NI biosensor. The target viruses are sandwiched by antibody-modified magnetic particles and optical markers. Using magnets and near-field illumination, the complexes, including the target viruses, are detected as moving optical signals.

Figure 4 shows a detailed schematic diagram of the detection chamber of the EFA-NI biosensor. The sensor chip is mounted on a trapezoidal prism. An s-polarized parallel light is incident on a trapezoidal prism at an angle parallel to the chip surface. In this setting, the angle of incident light on the surface of the sensor chip, which serves as a reflective surface, is designed to be greater than or equal to the critical angle. The incident light is totally reflected at the surface of the sensor chip, and the vicinity of the sensor chip surface is illuminated by the near-field light.

For the electric field enhancement of near-field light, the surface plasmon resonance based on a thin metal film on the surface (a leaking electric field with a height of approximately 100–200 nm) is often used for immunosensors. However, in the detection area up to about 200 nm in height, the detection target easily leaves the detection area with the EFA-NI biosensor that does not capture the detection target on the surface. Therefore, to use a wider space as the detection area, we adopted a waveguide mode excitation mechanism using a multi-layered waveguide, which is capable of forming a highly enhanced electric field with a height of approximately micrometers.

A mixture that included the sample, the antibody-modified magnetic particles, and the antibody-modified optical markers was introduced into the liquid cell formed on the sensor chip. The targeted virus was detected as a moving optical signal by applying a magnetic field from a sandwiched complex with the antibody-modified magnetic particles and the antibody-modified optical signal marker. Contaminants, which are noise sources, do not move in the same manner as magnetic particles when a magnetic field is applied, so optical signals originating from contaminants can be easily distinguished as noise.

We demonstrated the detection ability of the EFA-NI biosensor by detecting Norovirus Virus-Like Particles (NoVLPs) [42,43]. Norovirus is a pathogen that has been reported to be infected all over the world, and it is reported that there is a possibility of infection even if only about 20 virus particles are orally ingested [44,45]. Therefore, prevention of norovirus infection requires the detection of even very small amounts of norovirus in specimens suspected of viral contamination. The EFA-NI biosensor exhibits high detection ability in the range of 10^2^–10^4^ NoVLPs/mL (which the LOD ≈ 1 order of magnitude smaller than other motion-based methods, e.g., Pt-nanomotors based method [39]) and can measure for samples containing rich contaminants, such as sewage-treated water, even without a washing process [40,41]. Due to its high contamination resistance, this technology is expected to be applied to environmental virus detection.

## 3. Digital Bioassays Technologies in Virus Detection

**Figure 5:**
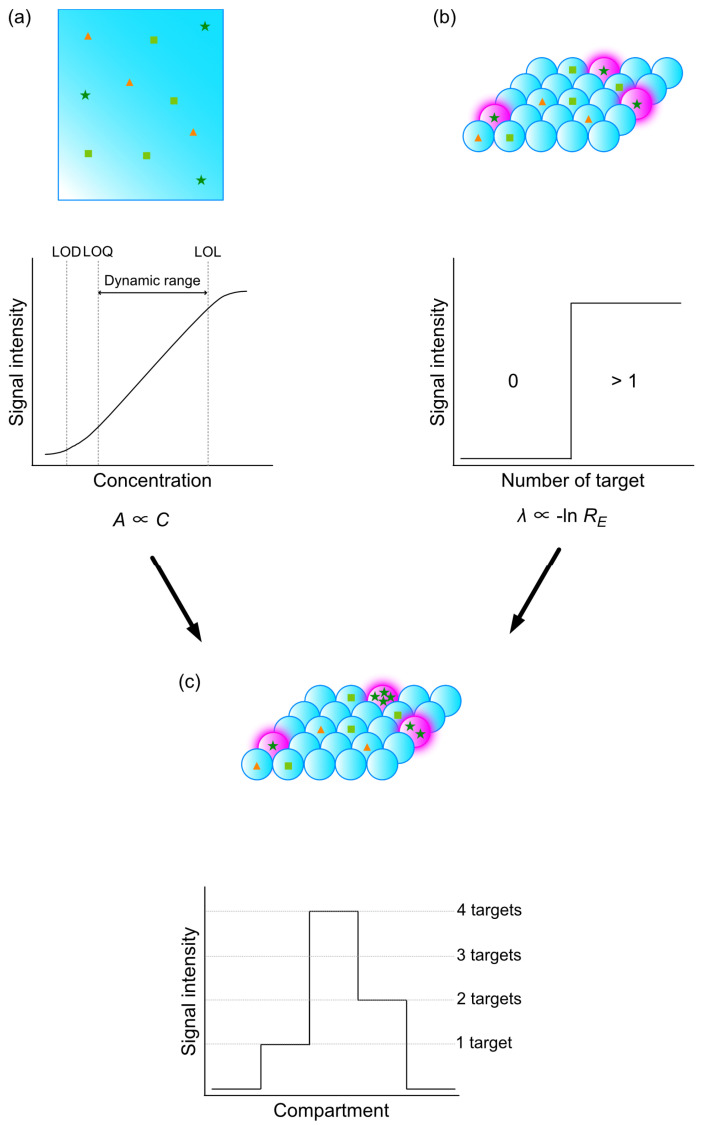
Digitalization of bioassays. (**a**) Analog bioassay. The relative concentration of the target in the analyte is estimated by the relative intensity of the signal generated from the target. Signal intensity is correlated with a concentration within the dynamic range. (**b**) Digital bioassay. Bioassay is compartmentalized either by microwell array or a microdroplet so that each compartment contains either 1 or 0 copy of the target. Then, the presence of the target in each compartment was detected, and statistics were collected. Digital bioassays allow for absolute quantification of the target in the analyte. (**c**) Digital–analog bioassays. More than 1 target was encapsulated in each compartment. Next, both the presence of the signal and the relative intensity of the signal generated from a target in each compartment were used to quantify the number of copies of the target. ☆ indicates target molecule, while other shapes indicate non-target molecules. Magenta indicates signal to be detected, which has been emitted by label molecule after reacting with target molecule.

To this point, the discussions revolve around virus detection technologies based on bulk analysis in a single reactor with large volume (>10 μL). These methods are known as analogue assays, in which the concentration of target, C, is proportional to the relative intensity of readout signals, I (I∝C) (Figure 5a). When the concentration of target is low, the probability of detection and relative signal intensity reduced, which results in false negativity. Furthermore, the sensitivity of detection based on analogue assays is incompatible to the detection of disease at the early stages of disease development. For instance, patients in the first two weeks of human immunodeficiency virus (HIV) infection have concentration range of p24 capsid antigen from 50 amol/L to 15 fmol/L [46], while the LOD of analogue assays is >pmol/L [47], rendering it challenging for the diagnosis and detection of viral infection at an early stage. Therefore, there is an urgent need for the development of technologies for ultrahigh sensitivity detection system to realize early diagnosis and the detection of viruses at low concentrations.

Two methods have been proposed to address the issue of limited sensitivity of detection in bulk system: (1) increase sensitivity of sensors. However, the sensitivity of sensors is constrained by a trade-off among the physical principles of sensors and its fabrication process which has come to its limitations. (2) The compartmentalization of the reaction system to enrich the target molecules in each compartment, thus increasing signal intensity and probability of detection. Theoretically, all compartments with target molecules can be detected as endpoint entity and are thus categorized as positive and negative compartments. Then, the number of both positive and negative compartments will be collected and statistically analyzed. Absolute quantitation of target molecule is defined as the product of average number of target molecules per compartment, λ, and the number of positive compartments.

(1) λ=−ln⁡〖R_E〗

where R_E is the fraction of negative compartments. This method is known as digital assays in contrast to its analogue counterpart, for its nature of binary detection. Sensitivity of detection can be easily increased by reducing the volume of each compartment, which makes compartmentalization a more favorable and cost-effective method compared to increasing the sensitivity of sensors (Figure 5b).

Bioassay through compartmentalization was first introduced by Lwoff and Gutmann in their study of lysogeny of Bacillus megatherium in 1950 [48]. Lwoff and Gutmann detected the lysogeny through the disappearance of Bacillus megatherium in droplets, followed by the release of hundreds of bacteriophages. This method allowed for the quantitation of a fraction of lysogenic bacteria which produced bacteriophages with ultrahigh sensitivity, which led to the award of the Nobel Prize in Physiology and Medicine in 1965 [49]. Digital assays later extended from single cell analysis [48,50] to protein and nucleic acid analysis, which has in turn facilitated the development of ultrahigh sensitivity virus detection technology.

### 3.1. Digial Polymerase Chain Reaction

Nucleic acid analysis is one of the standards, and the most used method in virus detection in contemporary society. It was invented by Kary Mullis in 1983, commercialized as Thermal Cycler by Certus and Perkin-Elmer in 1987, and it came to replace the Northern blot developed in 1977 [51] as a common practice in RNA analysis. Today, reverse transcription-quantitative polymerase chain reaction (RT-qPCR) is the gold standard for nucleic acid and virus quantitation (For a detailed review of application of RT-qPCR in virus detection, the readers are advised to refer to reference, e.g., [52]). While RT-qPCR has high dynamic ranges (>9 log), a low operating cost, and supports multiplexing, a complicated optimization process is required for qPCR to obtain reliable and reproducible results [53]. The sensitivity was low (≈10^6^ copies/mL), and its property as a tool for relative quantitation has resulted in large interlaboratory variation (≈27%) [54]. Therefore, there was an urgent need for a new method for nucleic acid analysis which could reduce the error of RT-qPCR and act as a reliable method for nucleic acid analysis.

Combining the methodology of limiting dilution and compartmentalization, as proposed by Lwoff and Gutmann, Vogelstein and Kinzler developed a method which could detect the presence of specific DNA fragment as either negatives or positives in 1999, which they named the method “digital PCR (dPCR)” [55]. As the target DNA is limiting diluted, the probability distribution of target DNA detected in each event was approximate to Poisson distribution (Equation (2)) [56],

(2) P(n)=(λ^ne^(−λ))/n!

where n is the number of molecules detected in each event. The absolute number of copies of DNA approximates the product of DNA in each compartment and the fraction of positive compartment. Apparently, when each compartment contains only 1 copy of DNA or no DNA, the sensitivity of the system is limited by the number of compartments screened. For instance, Vogelstein and Kinzler noted that by increasing the number of compartments from 96 to 1536, theoretical sensitivity in mutation detection could be reduced to ≈0.1% [55]. As we increase the number of compartments formed, the sensitivity will be limited only by polymerase error. By increasing the compartment to 20,000 to 30,000 compartments, various studies have shown that dPCR is more sensitive than RT-qPCR in virus detection under the same experimental conditions [57,58].

Today, there are generally two approaches in which compartmentalization was conducted: (1) chip digital PCR (cdPCR) based on microwell array, and (2) droplet digital PCR (ddPCR) based on microdroplets suspended in oil generated by microfluidic chips or vortex mixing.

**Figure 6:**
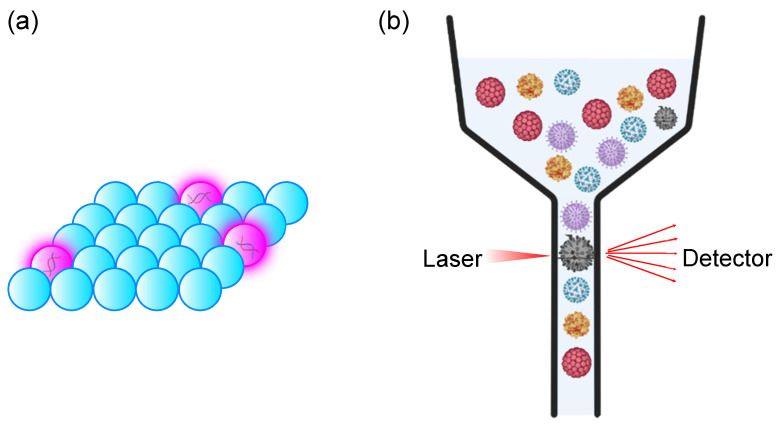
Digital bioassays for virus detection. (**a**) ddPCR assay. Target nucleic acid is amplified and detected in a compartment, which allows for high sensitivity of detection. The positive droplets are indicated as purple-overlayed droplets. (**b**) Flow virometry. Scattering of the laser was used to detect the presence of a virus in an analyte. Some patterns of particles are used to indicate various viruses.

Both approaches have been commercialized. The Fluidigm BioMark system and Thermo Fisher Quantstudio 3D are commonly used systems for cdPCR; Bio-Rad QX100/200, the RainDance RainDrop system, and the Stilla Naica system are commonly used ddPCR systems (Figure 6a).

Technically, cdPCR has larger compartment volume compared to ddPCR as the cost of fabrication increases exponentially with the reduction in size of the microwell. ddPCR also offers higher dynamic ranges (5–6 log) compared to cdPCR (2–5 log), and sensitivity for rare mutation detection. RainDance RainDrop dPCR system could detect 0.071 copies/μL [59], while the detection limit of Thermo Fisher Quantstudio 3D was 1.51 copies/μL [60], which is 3 times greater than the Bio-Rad QX100/200 ddPCR system (0.5 copies/μL) and 21 times greater than the RainDance RainDrop dPCR system. ddPCR has gradually become a more popular option compared to cdPCR for its lower operation cost and higher sensitivity. Nonetheless, both dPCR platforms are capable of performing direct quantitation of viruses without nucleic acid extraction [61], which could effectively prevent the loss of information in the nucleic acid extraction process.

Although the method demonstrated by Vogelstein and Kinzler relied on limiting dilution, it is unnecessary for digital assays. To ensure a sufficient number of empty compartments for quantification described in Equation (1), the range of λ is usually maintained within the range of 0.001 to 6 [62]. In other words, a maximum of six targets can be encapsulated in each compartment to conduct digital assays. By combining digital and analogue assays, i.e., setting the threshold value for signal intensity in each droplet corresponded to 1–6 copies of target DNA encapsulated and collecting the statistics of positive droplets, it is possible to counteract the consequence of encapsulating more than 1 copy of DNA in each droplet, which will further increase the accuracy of quantitation (Figure 5c).

A concern that remained for ddPCR compared to cdPCR is the volume uncertainty of droplets. Unlike microwell array, the volume of droplets is not constrained by the volume of container, and therefore there is higher volume variation in droplets (2–8%) compared to the microwell array (<1.0%). To address this issue, Pinheiro et al. have revised an equation (Equation (3)) which could counteractively correct the result of measurement with volume uncertainty [63].

(3) u=K√((u_c)^2+(u_V/V)^2)

where u is the uncertainty of target in droplets, K is the coverage factor ranging from 2.05 to 2.18 for 95% confidence interval, u_c is the uncertainty of compartment, u_V is the uncertainty of volume of compartment, and V is the mean volume of compartment. From Equation (3), we can note that uncertainty decreases as the number of compartments increases. It is possible to reduce the influence of uncertainty by increasing the number of compartments generated and analyzed.

With the recent development of vortex ddPCR [64], the cost of ddPCR can further be reduced as the requirement for specialized equipment and apparatus is eliminated. Furthermore, our group has recently developed a hydrogel capsule-based dPCR method [65]. By encapsulating the PCR reaction assay into a hydrogel capsule (which is composed by hydrogel shell and sol core), we eliminated the use of fluorinated oil and surfactant to stabilize the interface of droplets without compromising the efficiency of ddPCR. The cost has been reduced by 4000-fold compared to ddPCR as both soybean oil and hydrogel were cheap alternatives compared to fluorinated oil and surfactant. Furthermore, higher fluorescence intensity was also obtained at endpoint measurement due to the better dispersity of gel capsule in soybean oil compared to fluorinated oil, as fluorinated oil is denser than water. This property is particularly useful to detect nucleic acid, which is difficult to amplify. These technologies might further increase the attractiveness of ddPCR and enable its employment in a laboratory with limited resources.

### 3.2. Flow Virometry

When a portion of suspension is compartmentalized into a vast amount (10^2^–10^6^) compartment, statistic collection becomes a cumbersome task. Therefore, it is important to combine high-throughput analysis technology with digital assays for the practical application of digital assays. Before the advent of microscale analytic systems, various methods were developed to quantify colloidal particles aligned as single particles in fluid flow, based on photoelectric effect [66], electrical impedance [67], and fluorescence [68]. Combined with technology for the electronic separation of cells [69], these methods have contributed to the development of fluorescence-activated cell sorting (FACS) [70]. FACS generally employ a large suspension buffer-to-particle ratio to ensure that only a single particle is analyzed in each measurement (event).

With recent advances in FACS technology, which have allowed for the detection of particles size ranging from 100–1000 nm, it became possible to detect viruses with FACS. The first attempt to detect viruses and bacteriophage with FACS dated back to 1979 when Hercher et al. developed a custom flow cytometer for virus detection. By focusing the core of the stream to 2–20 μm and magnifying the laser with microscope magnifiers, the scattering signal from T2 bacteriophage (70–200 nm) and reovirus (60–80 nm) could be distinguished from background noise [71]. By changing the position of photodetector from 0.5°–15° to 15°–70°, a higher intensity of signal can be observed as forward scattering, which reduces the detection limit from μm order to 50 nm [72]. Although this technology is not informative, i.e., it did not provide much information about the virus, nevertheless, it is a breakthrough which has opened a new field of study known as flow virometry (Figure 6b).

The progress of flow virometry has been accelerated by the development of stable nucleic acid stain, which allows for the labelling and identification of DNA viruses [73]. The invention of bead-based assay (as discussed in Section 2) has allowed for the enrichment of viruses with microparticles and for te detection of viruses bound on microparticles [74,75,76]. By coating specific lectins on the surface of beads and employing beads containing fluorophore with different wavelengths, it is possible to identify viruses with viral glycoprotein on their surface besides the quantitation of virus particles.

Furthermore, the invention of flow virometry has also changed the paradigm of virus’s characterization. It has always troubled researchers and health workers that viral stocks generally contain large fractions of defective viruses compared to infectious viruses. Analogue assays usually measured the sample as bulk, which does not provide useful information on the composition of proteins in viruses. With flow virometry, El Bilali et al. demonstrated that it is possible to sort Herpes simplex virus 1 by infectivity based on tegument proteins VP16 and VP22 [77].

On the other hand, the development of microfluidic devices since the beginning of the 21st century has enabled customizable fluid manipulation in microelectromechanical systems. The successful generation of droplets in microfluidic devices [78] has allowed the integration of droplet-based methods invented by Lwoff and Gutmann [48] into this precise liquid manipulation system. With microfluidic, droplets at fL–nL scale can be generated at high-throughput, which has provided three great advantages for digital assays: (1) smaller compartment volume increases the relative concentration of samples in each compartments, thus the sensitivity of assays, and (2) smaller compartment size has reduced the field of illumination, thus, a brighter signal can be obtained from each compartment, (3) miniaturization increases the speed of reaction by l^2, where l is the length of reaction. To date, various sorting modules based on droplet microfluidics have been invented, which includes fluorescence [79], fluorescence lifetime [80], absorbance [81,82], scattering [83,84], Raman spectrum [85], X-ray [86], and UV-vis spectrum [87], which are particularly useful for virus sensing.

With the help of enrichment technology, e.g., bead-based enrichment [88,89], enrichment by specially designed probes [90], and droplets-encapsulating technology [91], the analysis of species of virus has become feasible. A virus can be captured, trapped, and lysed, and the nucleic acid can be amplified and detected, which has expanded the potential application of flow virometry.

### 3.3. Digital Enzyme-Linked Immunosorbent Assays

Protein analysis is another important approach for virus identification and quantitation. Among various methods for protein quantitation, ELISA has emerged as the golden standard for protein quantitation. As analogue assays, the LOD of analogue assays of ELISA is pmol/L. This LOD does not meet the sensitivity required for the early detection of a virus (several tens amol/L). Therefore, a single-molecule detection method with high sensitivity is required to detect proteins at amol/L. In 1961, Rotman reported a method to encapsulate single β-galactosidase molecule into emulsion and successfully measured the activity of individual molecules [92]. By observing the rate of change of fluorescence intensity in each droplet, Rotman discovered Poisson distribution in the trend, which is typical in digital assays. After several tens of years from Rotman’s pioneering work, digital enzyme assays using an array of microwells of femtoliter volumes for encapsulating enzyme molecules were reported [93,94,95], which provided the technical basis of single enzyme molecule detection.

**Figure 7:**
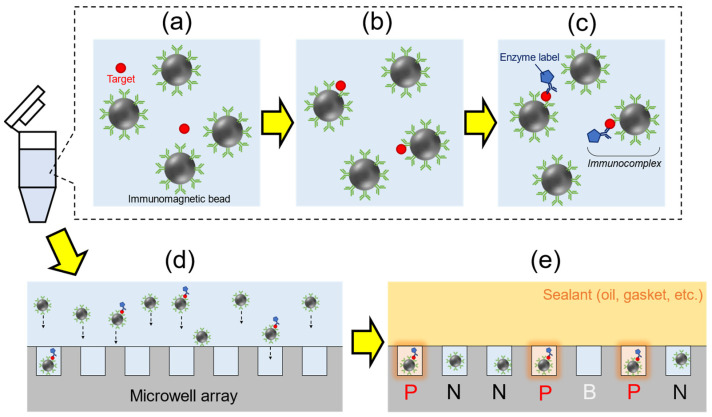
Schematic diagram of digital ELISA. (**a**) Before and (**b**) after capturing the target by immunomagnetic beads. (**c**) Formation of bead-target-enzyme immunocomplex. (**d**) Applying the immunocomplex and the beads to microwells. (**e**) Observation of sealed microwell array. P: positive well/bead, N: negative well/bead, B: blank well. The numbers of P and P + N are counted from fluorescence images and used to quantitate the concentration of the target.

Based on the technology of digital enzyme assays, Duffy’s group (Quanterix Corporation) has developed and reported the first digital ELISA (dELISA) system that employed targeted molecules capturing by immunomagnetic beads and enzyme labeling in 2010 [96]. The schematic diagram of dELISA is shown in Figure 7. First, an analyte containing target protein molecules was mixed with antibody-functionalized immunomagnetic beads (typically 3 μm in diameter) to capture target proteins in suspension (Figure 7a,b). Enzyme labels then bonded to the target molecules via antibody (Figure 7c). An immunocomplex containing antibody-functionalized magnetic beads, target molecules, and enzyme labels were formed. The immunocomplex was encapsulated into a microwell array (10^3^–10^5^ of wells with femtoliter volumes) with fluorogenic substrate (Figure 7d). When the immunocomplex is present in the microwell, fluorescence signal is generally generated through enzymatic reactions, and the statistic of positive wells was collected (Figure 7e). By using a microwell array which could only accommodate a single magnetic bead per well, e.g., a microwell with a diameter of 4.5 μm and a depth of 3.25 μm for a magnetic bead with a diameter of 2.7 μm [96], the ratio of the luminescent wells was equal to the ratio of the number of immunocomplex to total number of magnetic beads, which is used to evaluate the average enzyme number per bead. The first dELISA used approximately 50,000 microwells fabricated by processing optical fiber bundles, and a prostate-specific antigen (PSA) in serum was detected with the LOD of 200 amol/L (8 fg/mL) [96]. The result was 1000 times superior to the LOD of analogue ELISA (≈10 pg/mL for PSA). After the first report, the research group of Quanterix Corporation continued to develop digital ELISA-related technologies, including the dynamic range broadening of assays by using the average enzyme number per bead measured with digital and analogue hybrid methods [97], polymer-based microwell sensing plate with fluidic channel for automation of assays [98], and the theoretical analysis of digital assays [99], and commercialized the automated digital ELISA systems such as the Simoa series [100]. Simoa series has been widely used in viral protein detection, which includes the detection of HIV (LOD = 2.5 fg/mL in serum) [101], influenza virus (LOD = 0.59 fmol/L and 0.99 fmol/L for nucleoprotein and hemagglutinin in buffer solution) [102], and SARS-CoV-2 (20 fg/mL for N protein in nasopharyngeal swab) [103,104,105]. The sensitivity of dELISA is approximately 1000-fold higher than analogue ELISA. Also, several assays using CRISPR-based technology to target nucleic acids were established in a manner that mimics immunoassay detection systems such as immunochromatography [106]. Being a similar system to digital ELISA, CRISPR-based amplification-free digital RNA detection was recently developed to detect viral RNA (LOD = 5.7 fmol/L of SARS-CoV-2 RNA extract) using microwell arrays and CRISPR-Cas13 [107]. Besides, interferometric techniques can also be applied to the digital assay. Using interferometric scattering of the small particles, the size and count of the particles are visualized in the microscopic image [108,109]. By combining interferometry and sandwich immunoassay techniques, a commercialized device based on the principle of Single Particle Interferometric Reflectance Imaging Sensing has successfully detected extracellular vesicles (EV) with the LOD of 10^6^ to 10^9^ EV/mL [109].

dELISA technology flourish around the Simoa series, and various progress has been obtained, e.g., Kim et al. increased the number of microwell in dELISA by 1 order of magnitude, which lowered the LOD of dELISA to 2 amol/L [110]; Leirs et al. extended the application of dELISA from PSA detection to nucleoprotein detection [111]; dELISA without magnetic beads were developed by exploiting viral enzymes, e.g., neuraminidase of influenza virus [112]. The portability of dELISA was enhanced by using the camera of a smartphone and evanescent light illumination, which produced a portable dELISA system with 100 times higher sensitivity compared to rapid diagnostics kit [113]. The variation of dELISA with air as sealant instead of oil was also developed to enable solution exchange of microwells for performing multiple condition assays on the same target enzyme molecules [114].

Further studies have been conducted to improve the LOD of dELISA to zmol/L. While increasing the number of microwells is effective to improve LOD as a larger volume of suspension can be sampled [110], the field of observation has limited the maximum number of microwells to ≈10^6^ wells; reducing the number of magnetic beads could also improve the sensitivity of dELISA as it increased the fraction of immunocomplex analyzed, e.g., reducing the number of magnetic beads by 100-fold could improve sensitivity by 189-fold [115]. Nevertheless, low concentration of magnetic beads resulted in a longer reaction time for antigen capturing, which reduced the speed of detection [115].

Balancing the trade-off of sensitivity and speed of detection is a challenging task in dELISA with the current design, which allows for only single bead per well. A breakthrough is needed to achieve high sensitivity, such as rapid dELISA.

### 3.4. Multiparticle-Concentrated Digital Immunoassay

As mentioned above, dELISA for virus detection [101,102,103,104,105] achieves high sensitivity by combining the principle of digital detection and the efficient capture, concentration, and washing of targets with the help of magnetic particles. However, to increase the resolution of detection, it is preferable that only one magnetic particle is accommodated in a well. Therefore, it is desirable to have only one target bind to a magnetic particle. As dELISA measures viral proteins from lysed virion, it is difficult to know whether the virus particles maintain their particle shape, which may have a negative effect on the quantification of enveloped viruses, for their wide range of particle sizes and diversity in the number of proteins contained in each particle. In addition, the number of magnetic particles which could be used in dELISA is determined by the number of wells available. Even if more magnetic particles than the number of wells is used, they cannot be accommodated in the wells, which will increase the probability of false negativity. Therefore, it is difficult to speed up the capturing, concentrating, and washing processes by using high-concentration magnetic particles.

**Figure 8:**
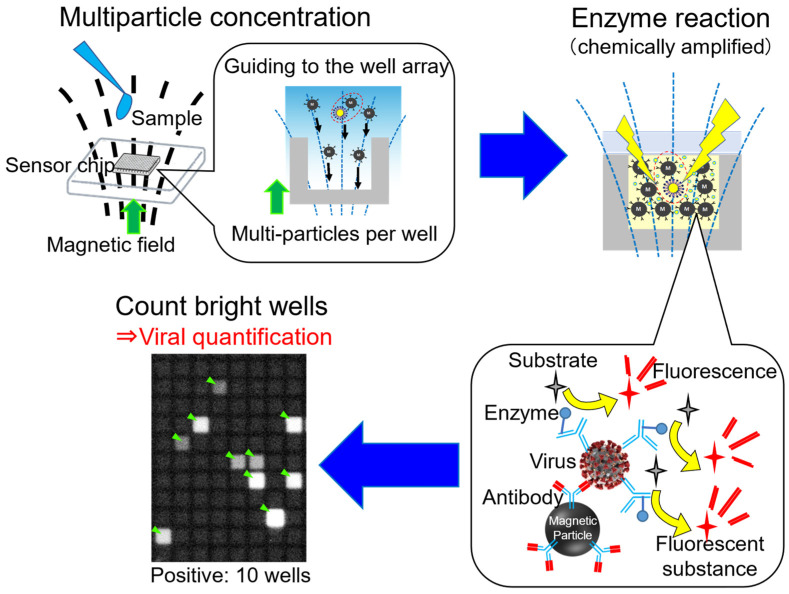
Schematic diagram of MCIDA. After capturing the targets by antibody-modified magnetic beads, bead–target–enzyme immunocomplexes are applied to microwells. Different from the digital ELISA, many immunocomplexes and beads are introduced in each microwell. After sealing and enzyme reaction, the sealed microwell array is observed, and we counted how many wells indicate fluorescence. Green arrows indicate the direction of the magnetic field. Yellow arrows indicate the reaction of the substrates changing to the fluorescent substance.

To address this issue, our group has proposed a lysis-free method that could simplify the complex and cumbersome sample preparation process, while achieving similar sensitivity to the PCR method, which we call a multiparticle-concentrated digital immunoassay (MCDIA) [116,117]. Figure 8 shows an outline of the detection principle of MCDIA. MCDIA uses digital detection technology, similar to the dELISA [101,102,103,104,105] which has been described above. The well array used consists of pL-sized holes which can accommodate many magnetic particles per well. Highly concentrated (>10^8^ particles/mL) antibody-modified magnetic particles can quickly capture viruses in the sample. Next, the enzyme-labeled antibodies are bound to the virus and unbound enzyme-labeled antibodies are removed. By rapidly guiding the complexes into the well array using a magnetic field, the viruses sandwiched with the magnetic particles and the labeled antibodies are concentrated in a short time. Almost all the particle–virus complexes and magnetic particles can be accommodated in the well array. Furthermore, they are enclosed in the well array together with fluorogenic substrates that react with the enzyme label. Fluorophore was released, and fluorescent signals are detected only in the wells, including the viruses. Viruses in the sample can be detected and quantified by counting the wells that emit fluorescent signals.

Theoretically, the MCDIA can achieve detection within a few to a few tens of minutes with a sensitivity equal to or greater than that of the PCR method. Both rapid virus-capturing and high sensitivity can be realized by introducing a multi-particle concentration method into the digital detection technology. We have successfully developed an influenza A virus (IAV) detection system [116] and a SARS-CoV-2 detection system [117] based on the MCDIA. Using the developed IAV detection system, the LOD of 100 copies/mL of IAV has been demonstrated, which is more sensitive than the PCR method, approximately 30 min from the mixing of the antibody-modified magnetic particles with the sample [116]. In addition, the SARS-CoV-2 detection system succeeded in detecting UV-inactivated SARS-CoV-2 equivalent to 100 TCID_50_/mL in a reaction time of 3 min, for which the concentration is approximately the lowest virus concentration (titer) that can be detected in the saliva of SARS-CoV-2 patients [118,119,120,121]. This indicates that this system can cover the clinical detection range. These demonstrations suggest that MCDIA is a high-performance system that could be used to detect low concentrations of viruses within minutes.

In future works, while maintaining sensitivity, we aim to shorten the entire detection time by automating and improving the efficiency of the procedure part and will achieve both the highest-level sensitivity and fastest-level sensitivity required for screening tests for asymptomatic people and for evaluating the risk of infection in a certain space. If a highly sensitive and reliable rapid on-site test is put into practical use, it is possible to control the spreading of viruses before it evolves into a pandemic by screening asymptomatic patients and reducing the risk of aerosol transmission. In the event of an infectious disease pandemic, we can also expect to avoid situations that cause huge social and economic losses, such as declaring a state of emergency or pre-emergency measures in the global pandemic of viral infection.

## 4. Pore-Based Sensing for Virus Particles Detection

**Figure 9:**
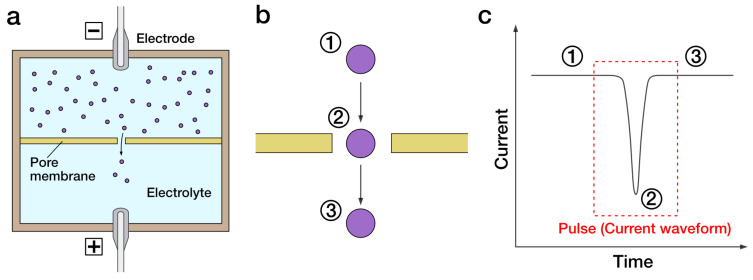
Schematic illustration of pore-based sensing. An electrolyte is divided by a pore membrane, and electrodes are placed on each (**a**). When voltage is applied to the electrodes, an ion current is generated that passes through the pore membrane, and the small particles (indicated as purple circles) in the solution pass through the pores by electrophoresis or electroosmotic force. Particles passing through the pore (One particle moves to ➀, ➁, ➂ in order) block the ion current (**b**), and the corresponding current value change is measured as a pulse (**c**).

Pore-based sensing is a promising candidate for the early detection of extremely small particles such as molecules, proteins, and viruses (50–200 nm). This method is known as “Coulter principle” or “resistive pulse sensing”. Pores usually consist of a nano- to micrometer hole within a substrate which forms a barrier between two electrolyte-filled reservoirs. A pair of electrodes are placed on each side of substrate, and voltage is applied to the pore and measures the ion current flowing through it (Figure 9). Particles can be counted in real time by modulation in an ion current derived from obstructing the pore in the translocation process. Since the magnitude of the ion current depends on the size of particles, pore-based sensing can measure the size of individual particles then output them as statistics such as transmission electron microscopy [122]. Notably, pores on the nanometer scale are called “nanopores” and are particularly suitable for measuring nanometer-sized viruses.

Nanopores are widely used in biochemical and single molecule detection, and could be made of biomacromolecule or synthetic materials. For example, molecular measurements using nanopores, in which α-hemolysin with cyclodextrins inside is embedded in lipid membranes, was reported [123,124], and pore-based sensing was recognized as a method for single molecule analysis. On the other hand, pore-based sensing using naturally occurring pores has limited the size of measured particles. Therefore, artificially fabricated pores have been developed for particle measurement. Viral particle sensing using artificially fabricated pores will be discussed in this chapter.

### 4.1. Fabrication of Pores for Sensing

An important aspect of nanopore measurement is techniques for making pores. There are many ways to penetrate thin membranes for the fabrication of pores such as ion beam drilling, electron beam lithography, electrochemical etching, etc. [125,126,127,128,129]. For example, it has been reported that the fabrication of pores in glass with sub-micrometer diameter uses a femtosecond-pulsed laser [126,130] to measure *paramecium bursaria chlorella virus* 1 with a diameter of 175–190 nm [131,132]. Other reports have also shown the use of a single heavy ion to fabricate pores [125,133,134], while in another method, a needle is used to penetrate a membrane [135]. This method is used for pore preparation, sold as a pore membrane for qNano and Exoid by Izon Science, and is a necessary process for tunable resistive pulse sensing (TRPS) (The details of TRPS are discussed below).

An advantage to using pores for particle measurement is that not only the size of the particles can be estimated, but also the waveform of electric current varies with the shape of nanoparticles. Therefore, the preparation of pores with precisely controlled geometry is also of great importance to obtain highly reproducible measurement. For example, precisely uniformed low-aspect ratio pore prepared by electron beam lithography and reactive ion etching can obtain the specific current waveform derived from the shape of measured particles [136,137,138]. Pore fabrication methods are developing according to the parameters of the particles to be measured.

### 4.2. Quantification of Virus Using Pore-Based Sensing

The conventional technique to measure viruses does not depend on the amount of virus particles but on infection titer such as hemagglutination inhibition assay and plaque assay, as viruses cannot be detected directly. PCR is also a conventional assay by measuring the number of viral nucleic acid, which can measure viruses quantitatively at first glance. However, viral integrity must be maintained for infectivity, and the detection of viral nucleic acid does not necessarily indicate risk of infection. Although the infectivity of the counted virus particles cannot be measured, the virus can be quantified from a different perspective than PCR because particles that have the shape of a virus can be counted.

A main challenge of pore-based sensing is that the detection opportunity depends on a stochastic factor, that is, it relies on the probability that a viral particle in the vicinity of a pore will pass through the pore. The frequency of passing particles increases with the density of particles. Therefore, this concentration range is the dynamic range of the pore sensor. Note that a passing efficacy is not only depends on a particle concentration but characteristics of particles. The main driving force for particles to pass through the pores is electrophoresis caused by the application of voltage. Accordingly, particles with higher or lower surface potential relative to their environment are more likely to pass through the pores, while particles with no charge rarely pass through the pores by electrophoresis. Electroosmotic flow also allows particles to pass through the pore [139], but the drive by electroosmotic flow is limited in scope. Therefore, the frequency of particles passing through is difficult to be directly treated as particle concentration.

The problem of pore-based sensing can be improved by external forces. Pore sensors, in which a pore is provided in a stretchable membrane and the size of the pore is changed by stretching, are called tunable resistive pulse sensing and are commercially available under the product names qNano and Exoid [140,141,142]. In the case of TRPS, the particles are forced to pass through the particles regardless of their surface charge because of the pressure-driven mechanism that allows the solution to flow through the pores. There have been reports of using TRPS to measure and evaluate the amount of vesicular stomatitis virus [143]. According to this study, the passing rate of viral particle is 1 particle per minute when 1.0 × 10^7^ particles/mL viral suspension was applied, which is roughly viral concentration at the limit of detection by single pore-based sensing. Although this value requires more virus than the PCR detection limit of 100 copies/mL, it should be considered that the viral particles are measured as they are without any manipulation, such as gene extraction or gene amplification. The fact that PCR involves dozens of amplification operations also indicates that pore-based sensing is inherently capable of detecting very small amounts of virus. Apart from this, the upper limit should also be considered. According to this report, the limit of linearity between counting rate and concentration of virus is approximately 1.0 × 10^10^ particles/mL viruses [143]. This is because high concentration of the virus results in miscalculation derived from temporary clogging, and the virus concentration must be adjusted for quantitative evaluation.

Pore-based sensing is a useful technique to measure the virus in the suspension quantitatively. Since the parameter of physical quantity of virus is essentially different from virus titer and number of nucleic acids, these different parameters should be matched at a viewpoint of infection risk. It is a challenging problem to quantify the risk of infection, and virus evaluation by pore-based sensing may be one tool to solve this.

### 4.3. Advanced Techniques of Pore-Based Sensing for Virus Detection

In the previous subsection, we discussed the property of virus counting quantitatively. However, an actual sample, including viruses, certainly has other impurities, which sometimes prevents a viral particle measurement. While pore-based sensing is an attractive method for evaluating each measured particle individually, the properties of the particles obtained are limited to physical parameters such as size and charge density. Therefore, pore-based sensing performance must be advanced to selectively measure biological particles such as viruses. Clogging of the pore, which is one of the most troubling problems for pore-based sensing, should also be solved, because the impurities contained in virus suspension could prevent the viruses from passing through the pore.

Hydrophilic plasma treatment is commonly used to prevent the clogging of pores. However, facilities for plasma irradiation are required, and the effect is lost over time. Surface functionalization of a pore is one effective technique to improve the performance of pore-based sensing. For example, preventing unwanted adhesion is a required technique in the field of biomaterials and biosensing, which is mostly solved by surface functionalization. It is known that polyethylene glycol can give the non-fouling character on a surface by preventing impurity approaches because of excluded volume effects derived from a hydrophilic chain [144]. Zwitterion-based materials are also effective candidates for preventing pore clogging. An adhesion mechanism is strongly related to the state of water condition around the materials, which is called bound water or non-freezing water [145], which promotes adhesion via the dehydration of bound water molecules. On the other hand, zwitterionic surface minimizes bound water and prevents adhesion [146]. It has been reported that the surface modification of zwitterions and polyethylene glycol to the pore inhibited pore clogging [147].

There are some methods for the specific detection of target particles. One technique is to use a specially shaped pore for sensing. As mentioned previously, a low aspect ratio pore can obtain a detail of particle shape based on current waveform. For example, several kinds of viruses, such as vesicular stomatitis virus, tobacco mosaic virus [148], bafinivirus, and ronivirus, have distinctive shapes and can be easily distinguished by their waveforms. High aspect pores provide accurate volume information because the entire particle enters the pore, and shape information can also be obtained, although not as much as with low aspect pores [149,150,151]. However, many kinds of viral particles are near spherical shape, which makes it difficult to distinguish the virus species. Therefore, a further advanced technique is required.

**Figure 10:**
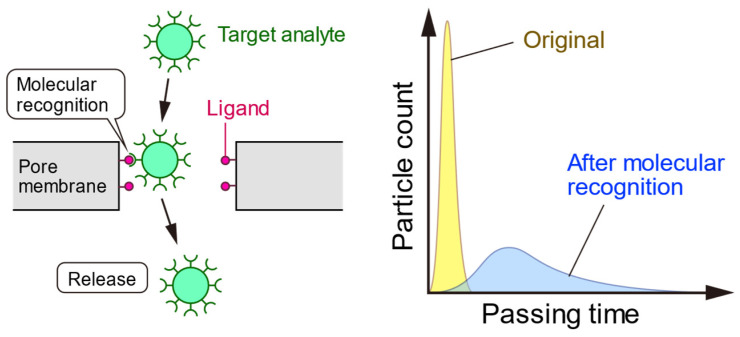
Molecular recognition on the pore surface. When a target analyte passed through the pore, molecular recognition prolonged the passing time by capturing the analyte on the pore. This translocation process has been recorded as a pulse waveform that can identify the target analyte.

Another approach is surface functionalization to capture the target. Conventional biosensors such as surface plasmon resonance (SPR) and quartz crystal microbalance (QCM) sensors use the ligands on the surface of the sensor for specific detection. Since ligands are provided on the surface of the pore or near pore and measuring the passing time of particles significantly reveals how the target is captured through molecular recognition (Figure 10), pore-based sensing has been reported not only for viruses but also for proteins [152,153,154,155,156,157,158]. On the other hand, the capturing of the target on pore surface has a risk of pore clogging because molecular recognition keeps the targets on pore surface, which results in congestion. Therefore, pore-based sensing by molecular recognition on the pore surface requires ingenuity in adjusting target concentration and binding strength.

**Figure 11:**
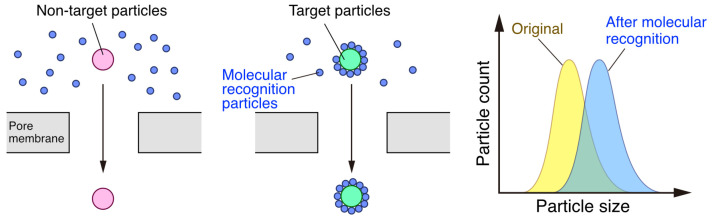
Molecular recognition using nanomaterials. If target particles exist in a sample solution, the target particles are covered with molecular recognition particles that shows the particle size changes (simultaneously, the signals change from ‘Original’ to ‘After molecular recognition’ as shown in the right graph of the figure).

Apart from this, the conjugation approach has also been suggested to identify the virus. For example, when the virus and antibody interact specifically, the volume of the virus increases because the virus is covered by the antibody due to complex formation. Therefore, the presence of the virus can be determined by changing the size of the viral particle [130]. It has been reported that artificial nanomaterials are used because they undergo significant size changes upon compositing (Figure 11). In this case, human influenza virus specific ligands are immobilized on 20 nm of gold nanoparticles, which can be used as virus recognition nanoparticles [159]. Since the typical size of influenza virus is 80–120 nm, the binding of nanoparticles gives the statistical change of virus particle size.

As a recent trend, artificial intelligence (AI) technologies for pore-based sensing have been investigated [139,155,157,160,161]. In this approach, the AI identifies the virus species by learning the characteristics of the pulse shape measured from each virus [160]. It is reported that AI techniques can identify not only the virus species but also the virus subtype such as influenza A H1N1 and H3N2 [139]. Although AI-based virus identification is a promising technology, it is important to note that this approach is not based on biological analysis such as traditional detection methods. It is difficult to explain on what basis the AI identifies the type of virus, so careful discussion is needed for its use as a diagnostic method. The combination of molecular recognition and AI are also investigated [155,157]. As mentioned above, if target particles bind strongly on the pore surface, serious pore clogging will occur easily. However, as the interaction is weakened, the difference in pulse shape becomes smaller, making simple identification more difficult. Therefore, AI technology capable of identifying subtle differences in pulse waveforms would be useful.

In this chapter, pore-based sensing technologies for virus detection have been discussed. This method is still an emerging technology and needs further development to become a general sensing technology. However, the method of detecting and analyzing viruses in their particle form is unprecedented and is expected to lead to rapid diagnosis. Since pore-based sensing only measures current values, it does not require optical devices, etc., and can be miniaturized. It is anticipated that pore-based sensing will be widely used as a particulate measurement method in the future.

## 5. Conclusions

The COVID-19 pandemic has caused tremendous damage to society and the economy around the world. While we hope that the tragedy can be prevented, unfortunately, the development of human society with dense traffic and the nature of cross-species virus transmission through random mutation [162] has dictated that the emergence of human viruses is inevitable. Therefore, it is important to continuously establish novel virus detection technology which could achieve high sensitivity and detecting speed, in addition to low cost and easy implementation to the society to prevent the development of virus emergence into a pandemic.

In this article, we provided an overview of various virus detection technologies (Table 1). As shown in Table 1, in general, conventional sensing technologies were limited by their capability to achieve both high sensitivity and high speed. Among these technologies listed in Table 1, we focused on some of them, applying three approaches using μm- or nm-scale components, i.e., bead-based assays, digital bioassays, and pore-based sensing technologies. They are potential technologies that could simultaneously achieve high sensitivity and rapid testing. Bead-based assays and digital bioassays (except flow virometry) are mainly based on chemical techniques, while pore-based sensing and flow virometry are methods based on physical approaches. In the chemical approach, the research community has focused on developing the methods to recognize the molecular fingerprints of viruses, i.e., (1) reagents for virus capturing with high specific recognition performance and (2) carrying out the amplification process until the absolute amount exceeds the lower limit of detection to increase the number of target or signal substances. To achieve the above-mentioned aim, two components are essential. Molecular design with high specific recognition is essential for aim (1), but practical measures are also necessary to increase the collision frequency between the target and the reagent. Also, in aim (2), the key lies in the method to improve the efficiency of the amplification process. Bead-based assays have the advantage of increasing the surface area to volume ratio for detection surface and the disadvantage to increase surfaces causing non-specific adsorption of the markers. Overcoming the issue of non-specific adsorption, bead-based assays could be implemented in rapid diagnostic for point-of-care multiplex virus detection. Digital assays have the advantage of absolute quantitation of viruses without the need for a calibration curve; the disadvantage is its need to divide samples quickly and efficiently. Overcoming the issue of sample loss and high-speed dividing, digital assays would be the optimal point-of-care virus detection methods.

On the other hand, physical approaches focus on the measurement of native signals generated from viruses, e.g., light scattering and electrical impedance. The label-free nature of physical approaches has made them an attractive approach in virus detection, as reagents to label viruses, e.g., antibodies are not basically necessary. However, it should be noted that viruses are not completely isomorphic and have a wide range of physical properties. Therefore, physical approaches exhibit less specificity compared to chemical approaches. For the reason above, the advanced techniques such as molecular recognitions and machine learning technology, mentioned in the relevant section, might help to improve the specificity of virus detection based on physical approaches by extracting and clustering the signal pattern specific to certain types of viruses.

In recent years, the introduction of digital measurement technology for virus detection has contributed to increasing the effective SNR of signals and reducing the LODs. In addition, the practical application of an extreme imaging device that realizes single photon counting has been announced [163,164]. The device may bring more than an order of magnitude improvement in the SNR compared to a conventional image sensor, which suggested that the reaction time of assay and detection time of signal can be further shortened. Furthermore, as can be understood by comparing one-dimensional measurement systems such as flow cytometry with two-dimensional measurement systems such as microwell arrays, it is also effective to improve throughput by increasing the dimensionality of the specimen.

In this way, various technological innovations have been boosted in recent years. By combining the innovations of these technologies, there is no doubt that the ideal virus testing method, which has both high enough detection sensitivity equivalent to that of PCR and quick testing time such as within 1 min, would be established in the near future.

## Figures and Tables

**Table 1:** The list of viral detection assays introduced in this review summarizes the characteristics of targets, required time, LOD, and quantitative.

Type of Assay	Targeted Element	Required Time	Limit of Detection		Quantitative?	Related Section
Nucleic acid amplification [15,16,17] (PCR, LAMP, NEAR, etc.)	Nucleic acid	1~4 h ^(a)^ Rapid type [22]: 10~20 min	100~1000 copies/mL (~aM level) Rapid type: 3000~20,000 copies/mL		Yes	1
CRISPR-based [106]	Nucleic acid	25~90 min ^(a)^ SATORI [107]: 5 min +RNA extraction time	3000~50,000 copies/mL SATORI: 3,000,000 copies/mL		Partially yes	3.3
Bio-barcode [32,33]	Protein Nucleic acid	<1.5 h	3 aM		Yes	2.3
dPCR [55]	Nucleic acid	5 h	1 copy (theory) 0.5 copies/μL (considering noise)		Yes	3.1
Flow virometry [71,72,73,74,75,76,77]	Viral particle Nucleic acid Protein	N/A; Volume, concentration, and flow cytometer dependent	1 particle/analysis		Yes	3.2
Pore-based [143]	Viral particle Protein	10–20 min	1 × 10^7^ particles/mL		Partially yes	4
Conventional immunoassay [18,19] (ELISA, CLEIA, Immunochromatography (IC))	Protein	ELISA, CLEIA [19]: 20 min~4 h ^(a)^ IC [18]: 10~20 min	ELISA, CLEIA [19]:	fM~pM ^(a)^	ELISA, CLEIA [19]: Yes IC [18]: No	1
			IC [18]:	~pM ^(a)^		
Bead-based ELISA [24]	Protein	3 h	0.001 RU/mL		Yes	2.1
Bead-based electrochemical [27]	Protein	2 h	10–11 IU/mL		Yes	2.2
EFA-NI [40,41]	Protein Viral particle	~30 min	~100 viral particles/mL (VLP detection)		Partially yes	2.4
dELISA [110]	Protein	5 h	10 zM		Yes	3.3
MCDIA [116,117]	Protein Viral particle	~30 min	~100 copies/mL (Not lysised virus)		Yes	3.4
Infectious viral titer [12,13]	Active virus	Plaque [12]: ~4 days TCID_50_ [12,13]: ~4 days	Plaque: >1 pfu/loading volume ^(a)^ TCID_50_: >0.5 TCID_50_/loading volume ^(a)^		Plaque: Yes TCID_50_: Yes	1

^(a)^ It significantly varies depending on the protocols and targets.

## Data Availability

Not applicable.
